# Collective dynamics of repeated inference in variational autoencoder rapidly find cluster structure

**DOI:** 10.1038/s41598-020-72593-4

**Published:** 2020-09-29

**Authors:** Yoshihiro Nagano, Ryo Karakida, Masato Okada

**Affiliations:** 1 Department of Complexity Science and Engineering, The University of Tokyo, Chiba, 277-8561 Japan; 2 Research Fellow of the Japan Society for the Promotion of Science, Tokyo, 102-0083 Japan; 3 Artificial Intelligence Research Center, National Institute of Advanced Industrial Science and Technology, Tokyo, 135-0064 Japan

**Keywords:** Computational neuroscience, Dynamical systems, Mathematics and computing, Information technology

## Abstract

Deep neural networks are good at extracting low-dimensional subspaces (latent spaces) that represent the essential features inside a high-dimensional dataset. Deep generative models represented by variational autoencoders (VAEs) can generate and infer high-quality datasets, such as images. In particular, VAEs can eliminate the noise contained in an image by repeating the mapping between latent and data space. To clarify the mechanism of such denoising, we numerically analyzed how the activity pattern of trained networks changes in the latent space during inference. We considered the time development of the activity pattern for specific data as one trajectory in the latent space and investigated the collective behavior of these inference trajectories for many data. Our study revealed that when a cluster structure exists in the dataset, the trajectory rapidly approaches the center of the cluster. This behavior was qualitatively consistent with the concept retrieval reported in associative memory models. Additionally, the larger the noise contained in the data, the closer the trajectory was to a more global cluster. It was demonstrated that by increasing the number of the latent variables, the trend of the approach a cluster center can be enhanced, and the generalization ability of the VAE can be improved.

## Introduction

Research on deep generative models, which extract essential features from an unlabeled dataset, is currently an active research area. Deep generative models have been reported to be useful in a broad range of applications, including generating images^1–4^, movies^5–7^, and text^8–10^. In particular, the conventional bidirectional network structure for the recognition and generation of images has made it possible to eliminate noise from cluttered images and smoothly interpolate between different images. Recognition is the process of mapping a data point to a latent variable, and generation is the inverse of this process.

Several studies have qualitatively highlighted the importance of repeating inferences between data space and latent space multiple times^1,2,11^. In the present study, repeated inferences are defined as a process by which a deep generative model repeats the recognition and generation of images. It was shown that by using noise-containing images as initial values, deep generative models can eliminate noise by repeating recognition and generation several times^2^. Moreover, compared to generating an output image from latent space to smoothly morph one image into another, repeating inferences several times improves the quality of the output image^11^. However, most of these studies only qualitatively evaluate output data through a one-shot inference from the latent space to output data. To fill this gap in the literature, we quantified the dynamics of repeated inferences to investigate why repeating inferences are effective for a wide range of applications.

In many cases covered by deep generative models, the data distribution is concentrated in the low-dimensional sparse subspace of the high-dimensional observation space. For example, in the case of natural image datasets, most of the space formed by the entire image corresponds to an image in which each pixel value is randomly chosen, but it is not plausible for natural images. The deep generative models extract a low-dimensional subspace in such a high-dimensional space by nonlinear mapping using neural networks. Because various factors, such as noise in real environments, cause original data points to deviate from this low-dimensional subspace, we are interested in *how the dynamics of the activity pattern during the inference phase is drawn into the subspace formed by the original training data*. In this study, we clarify how the activity pattern during inference in deep generative models approach the subspace where data are concentrated. In particular, we focused on the dataset that has a cluster structure, which is typically seen in image generation tasks and exists widely in nature.

We numerically analyzed the collective behavior of repeated inferences in a variational autoencoder (VAE)^1,2^, which is a typical type of deep generative model. We used the Modified National Institute of Standards and Technology (MNIST) dataset and Fashion-MNIST dataset, which are considered to have cluster structures consisting of 10 types of labels. We input noise-containing images to the trained VAE as initial values, and we numerically analyzed the transition of the activity patterns in the data space and the latent space. In particular, we calculated the time evolution of the distance to the vector in the latent space corresponding to quantify how the activity patterns approach the subspace of training data points, and we calculated the time development of the distance between the activity patterns in the latent space and the center of the clusters.

There are three major findings in our study. First, we numerically demonstrated that the dynamics of repeated inferences rapidly approach a center of the cluster in the latent space. Such transient behavior cooccurred with the perceptual refinement of the generated images in the data space. Second, by averaging all of these centers in the latent space, we considered the center of the cluster centers; by definition, training patterns, cluster centers, and the center of the cluster centers are hierarchically related in ascending order. We found that the inference dynamics approach the center of the cluster centers to the extent that the uncertainty of the input data increases due to noise. This result suggests that the model selects appropriate inference strategies in accordance with the fraction of the noise added to the input data. Third, we examined the effect of the latent variable dimension on inference behavior. As the number of latent variables increases, the internal representations of the clusters tend to become orthogonal, and the dynamics of repeated inferences approach each corresponding center. The generalization performance of the model was improved to the extent that the center of the cluster attracts the dynamics of repeated inferences. We also discuss the practical insight into the optimal number of inference steps from our experimental findings.

## Results

In the following, we numerically analyzed the dynamics of repeated infernce of VAE. A VAE is a generative model consisting of two neural networks: an encoder and a decoder^1,2^. An encoder gives a mapping of data, such as natural images to a latent variable space, and the decoder gives an inverse mapping. We evaluated the denoising behavior of a VAE trained with MNIST dataset for noisy inputs. The number of units of latent variable was set to 100 unless otherwise noted. Please see the Method section for the detail of VAE, network architecture, training and inference procedure, and noise injection.

### Dynamics of inference trajectory: an approach to cluster centers

First, we show that the dynamics of the latent space activities in VAEs are rapidly drawn into a low-dimensional subspace. In this study, we define the following operations as repeated inferences:

1 $$\begin{aligned} {\varvec{x}}(t+1)&= {\mathbb {E}}_{ p_{{\varvec{\theta }}}({\varvec{x}} \mid {\varvec{z}}(t)) } {[} {\varvec{x}} ], \end{aligned}$$

2 $$\begin{aligned} {\varvec{z}}(t)&= {\mathbb {E}}_{ q_{{\varvec{\phi }}}({\varvec{z}} \mid {\varvec{x}}(t)) } {[} {\varvec{z}} ], \end{aligned}$$

where $q_{{\varvec{\phi }}}({\varvec{z}} \mid {\varvec{x}})$ is the encoder network that maps ${\varvec{x}}$ to the latent space activity ${\varvec{z}}$, and $p_{{\varvec{\theta }}}({\varvec{x}} \mid {\varvec{z}})$ is the decoder network that gives the inverse mapping. First, we add the noise with noise fraction *p* to an image of the training dataset, and we set the image as ${\varvec{x}}(0) = {\varvec{x}}_0$. By repeating the above two equations *T* times, we obtain the trajectory of the repeated inference on data space ${\varvec{x}}(t)$ and latent space ${\varvec{z}}(t)$. In this study, we call the processes that gradually infer the plausible image by the above update rule as repeated inferences. We numerically analyze the dynamics of the activity patterns in data space ${\varvec{x}}(t)$ and latent space ${\varvec{z}}(t)$ from the qualitative/quantitative point of view. Please see the Method section for the detailed procedure about the network definition, inference procedure, and noise injection.

**Figure 1:**
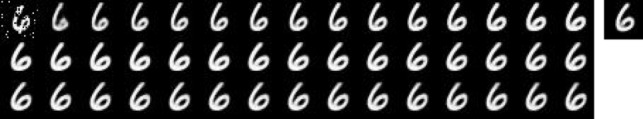
Consecutive samples in the data space (from left to right, one row after the other). The image of ‘6’ with $p=0.2$ noise applied was used as the initial value. The image ${\mathbb {E}}_{ p_{{\varvec{\theta }}}({\varvec{x}} \mid \bar{{\varvec{\xi }}}_{{6}}) } {[} {\varvec{x}} ]$ generated by the concept vector $\bar{{\varvec{\xi }}}_{{6}}$ is shown on the right

Figure 1 expresses the consecutive samples of the repeated inference in the data space. We used the MNIST database as the training dataset. The time development of the activity pattern in data space ${\varvec{x}}(t)$ is aligned from left to right, one row after the other. The upper-left image corresponds to the initial value, ${\varvec{x}}_0$. The image of ‘6’ with $p=0.2$ noise applied was used as the initial value. From the figure, the VAE removes the noise contained in the image in the first few steps and then gradually shifts to the specific image of ‘6’. We also show the consecutive samples for another image in Supplementary Information A.

**Figure 2:**
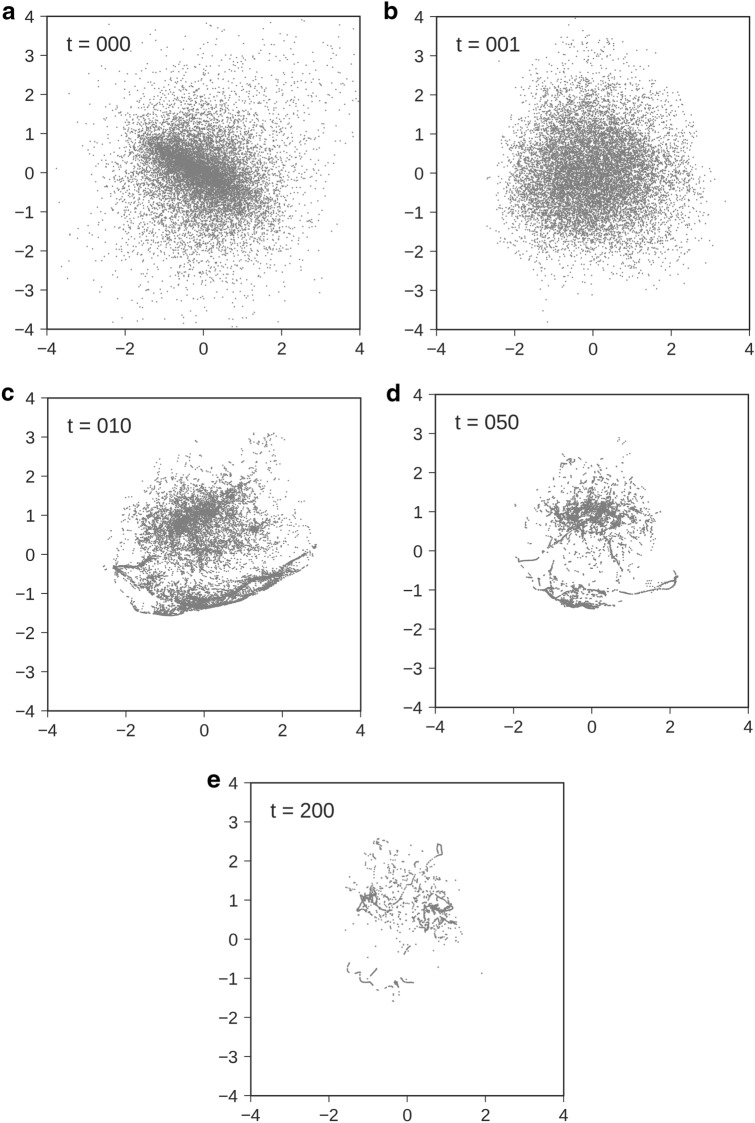
(**a**–**e**) PCA visualization of the VAE’s latent activity patterns. The *x*- and the *y*-axes represent the first and second principal components. Each figure corresponds to the snapshot of the activity patterns at time *t*. We used a different image of ‘1’ with a different noise realization as the initial value for each trial. The noise faction was set to $p=0.2$.

Then, we visualize the time development of the latent space activities during the repeated inferences. The temporal evolution ${\varvec{z}}(t)$ of the latent activity pattern for one initial image can be regarded as a trajectory in the latent space. Figure 2 shows the collective behavior of these trajectories in the latent space for multiple images. We used a different image of ‘1’ with a different noise realization as the initial value for each trial. We embedded the activity patterns in latent space into two dimensions using the principal component analysis (PCA). Because PCA has the degree of freedom of rotating eigenvectors, we performed PCA for the latent activity patterns included within $[t, t + \Delta t]$ for every *t* to stabilize the eigenvectors. Let *Z*(*t*) be the matrix that collects the activity patterns of the latent space within $[t, t + \Delta t]$:

3 $$\begin{aligned} Z(t) = \left[ \begin{array}{ccccccc} \vert &{} &{} \vert &{} &{} \vert &{} &{} \vert \\ {\varvec{z}}^{(1)}(t) &{} \cdots &{} {\varvec{z}}^{(N)}(t) &{} \cdots &{} {\varvec{z}}^{(1)}(t + \Delta t) &{} \cdots &{} {\varvec{z}}^{(N)}(t + \Delta t) \\ \vert &{} &{} \vert &{} &{} \vert &{} &{} \vert \end{array}\right] ^\top \in {\mathbb {R}}^{N \Delta t \times N_z}. \end{aligned}$$

We derived the matrix of eigenvectors $Q(t) = {[}{\varvec{q}}_1(t), \cdots , {\varvec{q}}_{N_z}(t)]$ for each time step:

4 $$\begin{aligned} Z(t)^\top Z(t) = Q(t) \Lambda (t) Q(t)^\top , \end{aligned}$$

and embedded the activity patterns in the two largest eigenspace as ${[}{\varvec{q}}_1(t), {\varvec{q}}_2(t)]^\top {\varvec{z}}^{(i)}(t)$. Each point in the figure corresponds to the latent activity pattern for the specific initial image. The *x*- and the *y*-axes represent the first and second principal components. At the beginning of the inference $t=0$ (Fig. 2a), the latent activities were widely distributed as one large cluster. These activity patterns branched into two clusters at $t=10$ (Fig. 2c). The first cluster was widely distributed in the upper part of the figure, and the second cluster formed a string-like distribution concentrated in the lower part of the figure. After that, the activity patterns converged to individual points or string-like regions. Especially at $t = 10$, the images generated for each of the two clusters were qualitatively different. The generated images of the lower cluster were ‘1’, while the images of the upper cluster tended to be the other numbers (see Supplementary Information B for the details). We also show the movies for these collective behaviors as Supplementary Materials.

From the aforementioned results, we numerically clarified that the latent activity patterns gradually branch into several clusters during repeated inference. Specifically, the collective behavior rapidly approached the low-dimensional subspace near $t=10$. We numerically quantify this type of approaching behavior. In the following, we assume that the dataset is composed of one cluster for each label for the clarity of numerical analysis. We now evaluate the distance between latent activity patterns and the center of the clusters.

5 $$\begin{aligned} \bar{{\varvec{\xi }}}_{\text {num}} = \frac{1}{N_{\text {num}}} \sum _i^{N_{\text {num}}} {\varvec{\xi }}_{\text {num}}^{(i)}, \end{aligned}$$

where ${\varvec{\xi }}_{\text {num}}^{(i)}$ indicates the activity pattern of the latent variable for the *i*-th training data with label $\text {num}$:

6 $$\begin{aligned} {\varvec{\xi }}_{\text {num}}^{(i)} = {\mathbb {E}}_{ q_{{\varvec{\phi }}} ({\varvec{z}}|{\varvec{x}}_{\text {num}}^{(i)}) } [{\varvec{z}}]. \end{aligned}$$

This definition is known as the mathematical quantity called “concept”, in studies on associative memory models^12,13^. The attraction of the activity patterns of neural networks into subspaces has been mainly studied with associative memory models^12,13^. In the problem setting of Matsumoto et al.^13^, they first randomly generated a small number of concept patterns, and then they made memory patterns with a precise correlation with these concept patterns. In other words, the concept pattern vector corresponds to the center of each cluster. We call $\bar{{\varvec{\xi }}}_{\text {num}}$ a concept vector and ${\varvec{\xi }}_{\text {num}}^{(i)}$ a memory vector for the *i*-th training data in the following sections. The relationship between the time development of inference and the concept vector of each label (‘0’–‘9’) represented in the MNIST data was numerically analyzed.

**Figure 3:**
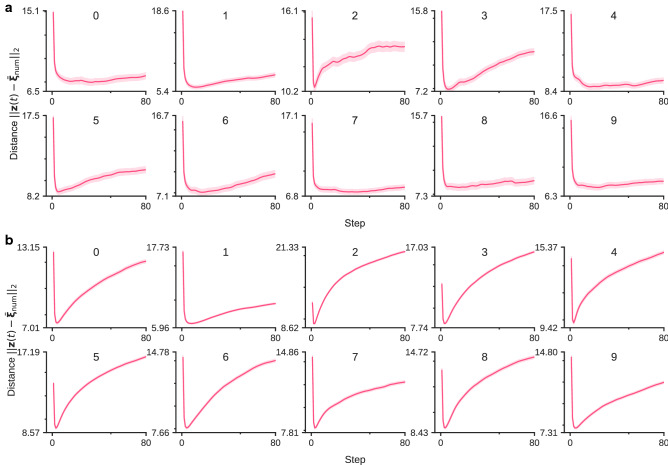
Time development of the Euclid distance for all labels of the MNIST data. The distances from $\bar{{\varvec{\xi }}}_{\text {num}}$ are shown in (**a**) and the distances from ${\varvec{\xi }}^{(i)}_{\text {num}}$ are shown in (**b**). The shades represent the $\pm 1$ standard error of the mean (300 trials). We used a different image of each label with a different noise realization as the initial value for each trial. All figures were generated with the noise fraction $p = 0.2$.

We show the image ${\mathbb {E}}_{ p_{{\varvec{\theta }}}({\varvec{x}} \mid \bar{{\varvec{\xi }}}_{{6}}) } {[} {\varvec{x}} ]$ generated by the concept vector of ‘6’, $\bar{{\varvec{\xi }}}_{{6}}$ on the right side of Fig. 1. By qualitatively comparing this image and the consecutive samples, it is suggested that the result of the VAE inference approaches the image generated by $\bar{{\varvec{\xi }}}_{{6}}$ once. Here, we define a trajectory “approaching to a concept vector” as follows: the trajectory whose distance to the concept vector takes a minimum at a unique halfway point and is closer than its synthetic linear interpolation. We quantitatively evaluated the gradual changes of the Euclidean distance; namely,

7 $$\begin{aligned} \Vert {\varvec{z}}(t) - \bar{{\varvec{\xi }}}_{\text {num}} \Vert _2, \end{aligned}$$

between the neural activity patterns and the cluster center for every label of MNIST data in the latent space (Fig. 3a). The distance between the cluster center and 300 different initial images was calculated. Each figure corresponds to each label, which was used as initial input for the VAE. The *x*-axis expresses the time step *t* of repeated inference, and the *y*-axis expresses the Euclidean distance (Eq. (7)). It was clarified that the trajectory of the VAE’s inference rapidly find the cluster structure. This result is qualitatively consistent with all labels of the MNIST data. A previous study using associative memory models^13^ reported that activity patterns approached the concept vector once in the middle of inference when the inference was started from data with noise applied to each memory pattern. Figure 3b shows the same figure for the distance between the activity patterns and the memory vector. As same for the concept vector, the activity patterns were closest to the memory vector early in the inference. If the concept vector is a stable fixed point, the activity patterns should monotonically approach the concept vector. In other words, these results suggest that the latent space of the trained VAE has a saddle point structure that attracts in the direction to which noise is applied and diverges in the orthogonal direction. The results obtained in this study were qualitatively consistent with these previous findings. Note that, the Euclid distance does not necessarily reflect the closeness of the measurement to the group if the clusters are not spherical. We examined this possibility in Supplementary Information C. We also performed the same numerical experiment on the Fashion-MNIST dataset^14^, which is a dataset of Zalando’s article images consisting of various fashion images. The numerical results for the Fashion-MNIST dataset were also qualitatively consistent with those for the MNIST dataset. Please see Supplementary Information D for more details.

### Relationship between data hierarchy and inference

We arbitrarily determined the amount of noise added to the initial input images in the previous section. To examine the effect of noise on the dynamics of repeated interferences, we then modulated the amount of noise. Because noise in input images causes the data to deviate from the original distribution, we created another class, the “abstract concept vector” for convenience, which averages all the labels’ concept vectors, as well as the concept and memory vectors. By measuring the distance between the trajectory of each neural activity pattern and its corresponding classes in the latent space, we identified the class that most attracts the neural activity patterns.

**Figure 4:**
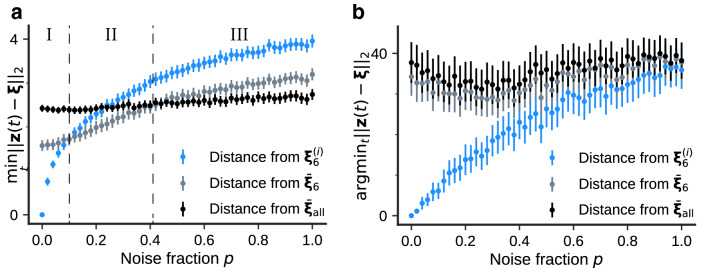
(**a**) Minimum distances from concepts according to noise fraction *p*. The bars represent the $\pm 2$ standard error of the mean (500 trials). We used a different image of ‘6’ with a different noise realization as the initial value for each trial. (**b**) Time step of which the distance takes a minimum.

We define the “abstract concept vector” as

8 $$\begin{aligned} \bar{{\varvec{\xi }}}_\text {all} = \frac{1}{10} \sum _{\text {num=0}}^9 \bar{{\varvec{\xi }}}_{\text {num}}. \end{aligned}$$

The three classes (memory vectors, concept vectors, and the abstract concept vector) are in a hierarchical relationship from detailed to coarse information in the order ${\varvec{\xi }}_\text {num}^{(i)}$, $\bar{{\varvec{\xi }}}_\text {num}$, and $\bar{{\varvec{\xi }}}_\text {all}$. Note that the abstract concept vector is not a useful representation from the practical viewpoint. We chose this metric as an anchor to unveil the dynamics of repeated inferences. We calculated the minimum distances between neural activity patterns ${\varvec{z}} {(}t)$ and corresponding classes,

9 $$\begin{aligned} \min _t \Vert {\varvec{z}}(t) - {\varvec{\xi }} \Vert _2. \end{aligned}$$

Figure 4a shows the minimum distances according to the noise fraction. In Fig. 4a, the *x*-axis represents noise fraction *p*, which is the probability that the image intensities of the pixels are swapped. For every noise fraction, the minimum distances between the firing pattern ${\varvec{z}} {(}t)$ and hierarchical concept vectors were calculated by changing the initial image 500 times. The dots in the figure express the mean of the minimum distance, and the bars are the $\pm 2$ standard error of the mean (500 trials). We divided the parameter regions into three stages, I, II, and III, corresponding to the minimum distance between the firing pattern ${\varvec{z}}(t)$ and hierarchical concept vectors, ${\varvec{\xi }}_{6}^{(i)}$, $\bar{{\varvec{\xi }}}_{6}$, and $\bar{{\varvec{\xi }}}_\text {all}$, respectively. In stage I, the firing activity was closest to the original pattern ${\varvec{\xi }}_ {6}^{(i)}$ with a small amount of noise. Interestingly, the closest class was $\bar{{\varvec{\xi }}}_{6}$ with moderate noise in stage II. The activity was close to the abstract concept vector $\bar{{\varvec{\xi }}}_\text {all}$ in stage III. In stages I and II, the memory was successfully retrieved because the inference path was close to the cluster in which the input data belonged; however, in stage III, the model could not determine the original cluster, so recall failed. Accordingly, the model achieves the inference dynamics depending on the input uncertainty. Figure 4b shows the time step of which the distance takes its minimum according to the noise fraction. The activity pattern approached the memory vector earliest in all stages. In addition, the time step of which the activity patterns are closest to the memory vector was dependent on the amount of noise. The dependence on the noise fraction of the minimum time step for the concept or the abstract concept vector was less significant than the one of the memory vector. These results suggest that the number of inference steps should be increased according to the amount of noise included in the input when performing noiseless reconstruction. On the other hand, the inference step should be around 20–30 independent from the noise fraction when performing label detection.

**Figure 5:**
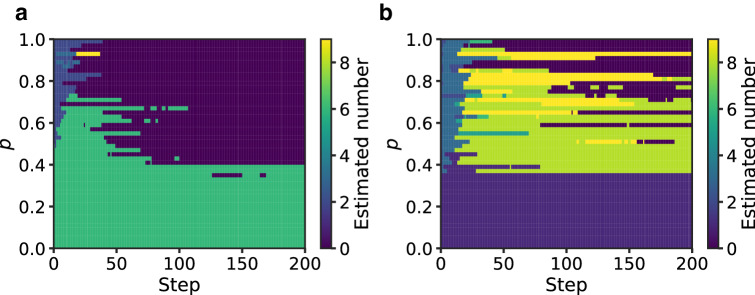
Estimated label of generated images. The class labels of the generated images at each step of inference were predicted using the classification network. The heatmap represents the label with the highest number of predictions in 200 trials. We used a different image of ‘6’ for (**a**) and ‘1’ for (**b**) with a different noise realization as the initial value for each trial.

To confirm the abovementioned suggestion about the label prediction, we estimated the class label of the generated images at each step of inference. Figure 5 represents the label with the highest number of predictions in 200 trials. We used another convolutional neural network as a classification network in each trial. The classification network has the following structure: Input-Convolution-Convolution-Pooling-Dropout1-FullyConnected-Dropout2-SoftMax. The kernel size of the convolution is three, the pooling size is two, and the dropout probability is 0.25 and 0.5 in order from the input side. We used a rectified linear unit (ReLU) as the activation function. The classification network was trained on the original MNIST dataset before classifying the generated images of VAE. Based on the Fig. 5a, the generated images started from ‘6’ were classified correctly in every time step in stages I and II ($p<0.4$). In stage III, the generated images were classified as ‘3’ or ‘4’ at the beginning of inference and were classified as ‘0’ at the end of inference. There was a particular region of inference steps that the generated images were ‘6’ for $p<0.7$, and the region was close to 20–30. The result for the generated images started from ‘1’ (Fig. 5b) was qualitatively consistent for the result of ‘6’, while the optimal range of inference steps was small or vanished against the middle amount of noise ($0.4< p < 0.7$). These results were also consistent with the above mentioned suggestion.

**Figure 6:**
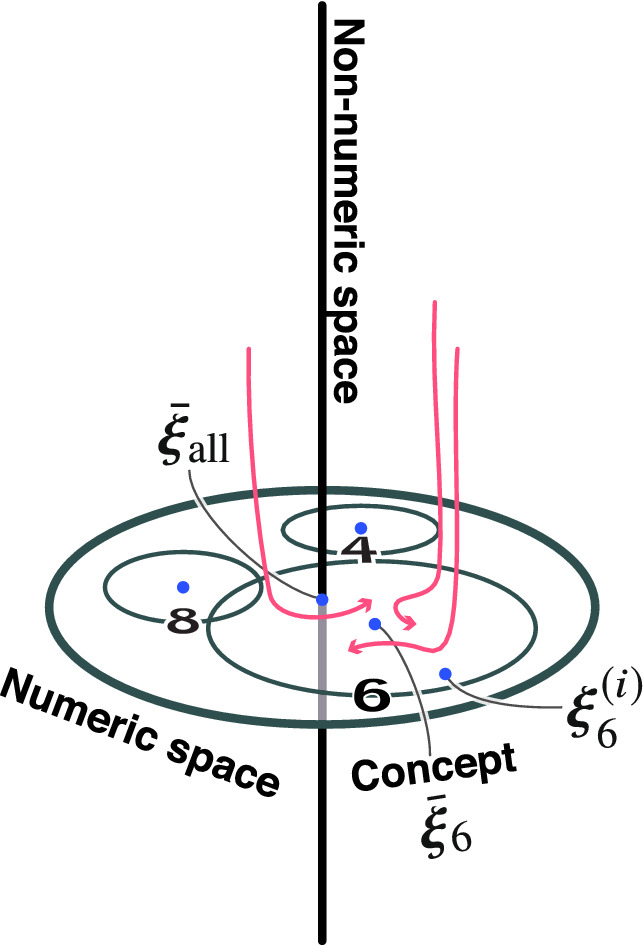
Schematic diagram of firing patterns in the latent state space.

**Figure 7:**
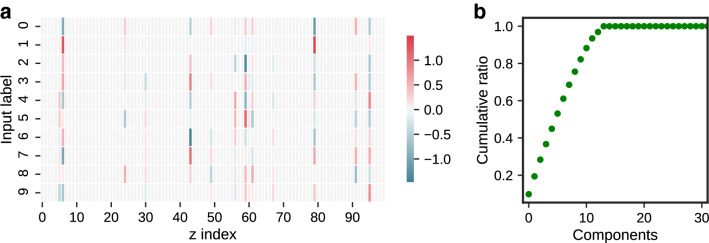
(**a**) Activity pattern in the latent variable space of each cluster center. The *x*-axis represents the neuron index of the latent variable, the *y*-axis represents the label, and the heat map shows the activity pattern of each neuron. (**b**) Cumulative contribution ratio of principal components.

As shown previously, the VAE extracts the cluster structures inherent in the MNIST data and infers images through the center of each cluster. These experimental results indicate that the dynamics of this inference are as shown in Fig. 6. As shown in Fig. 7a, when the dimensionality of the latent variable is large, only a few neurons contribute to representing the MNIST dataset. They would span a space that expresses each number (depicted as the numeric space in Fig. 6). According to the manifold hypothesis^15^, adding noises to images will cause the initial value to deviate from the numeric space, which is believed to be low-dimensional. The results of our first analysis suggest that when the inference begins with a position far from the space expressing the MNIST data, the activity patterns first approach the memory vector and then quickly go to the corresponding concept vectors.

### Effect of latent variable dimensionality

Because the VAEs are the generative models that learn the mapping between the high-dimensional data space and the low-dimensional latent space, the dimensionality of the latent space is the essential hyperparameter for acquiring internal representation. The setting of the latent space dimensionality is predicted to drastically affect not only the quality of generated images and generalization ability but also the dynamics of repeated inferences. In this section, we numerically analyze the effect of the latent space dimensionality on the dynamics of repeated inferences.

We first show the relationships between the cluster centers of each label for the aforementioned setting (Fig. 7). We set the dimension of the latent space to 100 in these experiments. The activity patterns in the latent variable space of each numerical concept are shown in Fig. 7a. The heat map expresses the activity pattern of each neuron, which corresponds to the latent variable, where the *x*-axis represents the hidden neuron’s index, and the *y*-axis represents the label. Only a few neurons out of 100 contribute to information representation, and many neurons are pruned and inactive. According to our observations, 14 out of 100 neurons were active. The dimensions of the latent space were examined using the cumulative contribution ratio determined by principal component analysis. The cumulative contribution ratios of each principal component when the training images were given to the VAE were shown in Fig. 7b. The variance in the latent space was explained entirely by 14 dimensions, and 70% of this was explained by nine dimensions. Then, we quantified how much the concept vectors on latent space, which correspond to the row vector of Fig. 7a, correlate with each other. We define the cosine similarity matrix *C*, where the element of the *i*-th row and *j*-th column is the cosine similarity between the concept vectors of labels *i* and *j*:

10 $$\begin{aligned} C_{ij} = \frac{\bar{{\varvec{\xi }}}_{i} \cdot \bar{{\varvec{\xi }}}_{j} }{ \Vert \bar{{\varvec{\xi }}}_{i} \Vert _2 \Vert \bar{{\varvec{\xi }}}_{j} \Vert _2}. \end{aligned}$$

**Figure 8:**
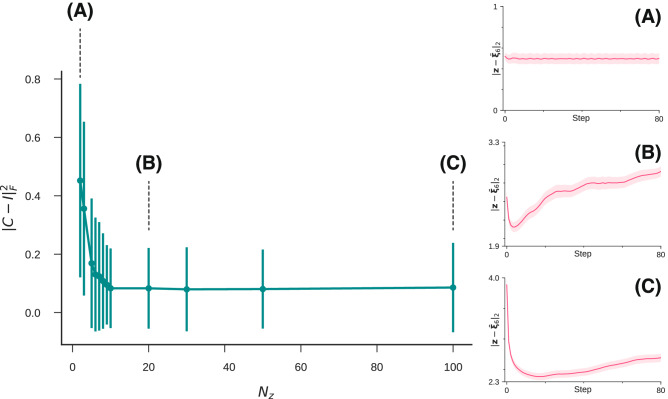
The orthogonality between the concept vectors in latent space. The error bars represent the $\pm 1$ standard deviation.

We quantified the orthogonality between the concept vectors as $\Vert C - I\Vert ^2_F$, where $\Vert A\Vert ^2_F$ is the Frobenius norm of *A*: $\Vert A\Vert ^2_F = \sqrt{\sum _{ij} A_{ij}^2}$. By definition, the cosine similarity between concepts of the same label is one. However, the cosine similarity between concepts of different labels in nondiagonal terms is minimal, namely, near zero. In other words, as the vectors between the labels are orthogonal, the above quantity approaches zero. The left side of Fig. 8 shows the aforementioned orthogonality according to the dimension of the latent space $N_z$. We trained VAEs from scratch for each $N_z$ and calculated the orthogonality for learned representations. From the figure, the orthogonality of the internal representation increased with $N_z$, and the orthogonality converged to a value close to zero when $N_z$ was approximately 10–20. We also visualized the typical dynamics of repeated inferences for $N_z=2, 20, 100$ on the right side of the figure as (A), (B), and (C). We used the same setting as that of Fig. 3 except for $N_z$, and the trained VAEs started repeated inferences from the images of ‘6’ with input noise. The approach to the cluster center mentioned above appeared remarkably with the increase in the orthogonality of the internal representation. Because previous studies on the associative memory models^12,13,16^ also identified an approach to the concept vector during inferences under the assumption that the concept vectors were orthogonal, our findings were qualitatively consistent with these studies. We also show the detailed values of the similarity matrix *C* for each $N_z$ in Supplementary Information E.

These results and the previous findings imply that orthogonality is necessary between cluster centers for the trajectory of inference to be drawn into the cluster center. Because the number of latent variables decreases, it is necessary to express data in fewer dimensions, and the orthogonality is lost. The reduction in the number of latent variables is considered to cause unstable memory patterns corresponding to the training data, and only the center of clusters is stabilized. As a result, the trajectory of inference goes straight to a stable point. We also numerically assessed whether other labels confuse repeated inferences in the VAE (e.g., although an inference starts from label ‘6’, it is incorrectly attracted to the concept associated with label ‘0’). The result of this assessment is shown in Supplementary Information F.

**Figure 9:**
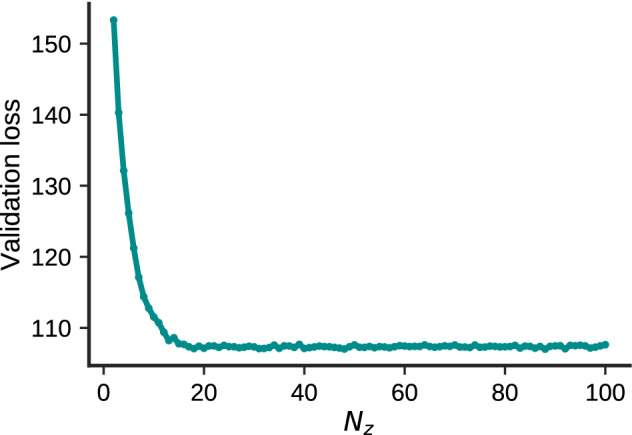
Generalization error for the number of elements of latent variables $N_z$. The *y*-axis represents the variational lower bound of the log-likelihood of the test data.

We also numerically analyzed the generalization performance according to $N_z$ (Fig. 9). The performance of the model was evaluated using the variational lower bound (Eq. (11)) of the log-likelihood for the test MNIST data. In each $N_z$, parameters that minimize the generalization error at epoch 100 with a total of nine conditions were selected from learning rates 0.01, 0.001, and 0.0001 and minibatch sizes of 50, 100, and 200. The generalization error was the minimum value in the vicinity of $N_z = 14$, and it did not change significantly afterward. In total, 14 of 100 latent variable neurons express training data under condition $N_z = 100$ (Fig. 7a), and the number of neurons that minimize the generalization error is consistent with this result. These results suggest that approximately 14 latent neurons are required to express the MNIST data in the network structure used in this study. Moreover, in the vicinity of $N_z = 14$, the cluster structure appears in the representation of the latent variable space, and the trajectory of inference is drawn into the concept. These results suggest that it is possible to judge the generalization performance of the model without computing the generalization error or orthogonality of internal representations by simply observing the dynamics of repeated inference.

## Discussion

In this study, we numerically analyzed the dynamics of repeated inferences in VAEs for the datasets with a cluster structure. Based on the numerical analysis of the collective behaviors, the activity patterns in latent space rapidly approached a specific subspace. We also found that VAEs extract the cluster structures inherent in the MNIST and infer images via the center of each cluster. The results of the first analysis suggest that when the inference starts from a point far away from the original data distribution, the repeated inferences approach the concept vector at high speed. The approach of activity patterns to the area where the training dataset is concentrated is considered to be the cause of the improvement in the quality of the generated image by repeated inference, which was perceptually noted in the previous research.

The learning and inference of multiple memory patterns have been widely studied using associative memory models^16–18^. In an associative memory model with multiple embedded, correlated patterns, the centroid of the correlated patterns spontaneously evolves to a fixed point^12^, and the time evolution of the activity patterns approaches the concept^13^. The results of our first and second analyses are qualitatively consistent with these findings, suggesting that the mechanism underlying the dynamics of repeated inferences in the VAE is related to the traditional associative memory model.

Previously, several studies demonstrated that repeated inferences successfully denoise^2^ and improve the quality of inferred images^11^. Our study suggests that the dynamics of repeated inferences approaching the center of the cluster inherent in the data lead to denoising and improving the quality of output images, which were quantitatively observed in the data space. It is critical to use a sufficient number of latent variables to precisely represent the concept inherent in the data; if the number of the latent variables is insufficient, the cluster structures will not be realized in the latent space, so the concept will be hardly identified. Our results suggest that stage II in Fig. 4a appears only when the number of latent variables is sufficiently large, and the number of latent variables qualitatively changes the dynamics of repeated inferences.

We also studied the time profile of repeated inference. Our numerical experiments revealed that the latent activity pattern, which started from noisy input, approached the noiseless embedding (memory vector) earliest. In addition, the time step of this approaching was dependent on the amount of noise. These results gave us the practical implication about the optimal number of steps of VAE’s repeated inference. The VAE can be used for several purposes, including noiseless reconstruction and embedding unknown data points for label detection. Our numerical experiments suggest that the number of inference steps should be increased according to the amount of noise when performing noiseless reconstruction. In addition, when performing label detection, the inference step should be larger than noiseless reconstruction.

In this study, we numerically analyzed the repeated inferences of VAEs for specific datasets. We mainly focused on the MNIST and the Fashion-MNIST, which have clear cluster structures. Hierarchical structures are one of the primary concerns of previous studies on the relationship between the structure of datasets and the behavior of deep neural networks. For example, deep neural networks are claimed to express abstract information in deep layers^19,20^. In particular, Bengio et al. stated that deep layers speed up the mixing of Markov chains using their ability to manifest abstract information. Moreover, Saxe et al. analytically showed that deep neural networks learn data in order from large to small modes, and the internal representations branch accordingly^21^. To clarify the universal behavior regarding the inference dynamics of deep generative models, we need to address the structure of various datasets that are not limited to the cluster structure, including the hierarchical structure.

Recently, researchers have been actively working on models that can capture features inherent in data as forms of internal representations^22–28^. The VAE used in this study embeds the data points in a simple isomorphic Gaussian distribution. As a next step to expand on these works using other deep generative models, we aim to further investigate what factors influence the behavior of repeated inferences approaching the concept. In addition, we will analyze the dynamics of repeated inferences in another model using training datasets with more and varied hierarchies.

## Method

Here, we first describe the summary of the VAE. Then, we describe the network architecture which we used in numerical experiments and how to train the VAE. Last, we describe about an inference procedure.

### Variational autoencoder

A VAE is a generative model consisting of two neural networks: an encoder and a decoder^1,2^. An encoder gives a mapping of data, such as natural images to a latent variable space, and the decoder gives an inverse mapping. The objective function of the VAE is obtained by finding the variational lower bound of log-likelihood $\sum _i \log p({\varvec{x}}^{(i)})$ for *N* training data $X=\left\{ \varvec{x}^{(i)} \right\}_{i=1}^N$. In the following, we consider a parameter ${\varvec{\theta }}$ that maximizes the log-likelihood $\log p_{{\varvec{\theta }}} ( {\varvec{x}}^{(i)} )$ at each data point. Using the latent variable ${\varvec{z}}$ and its conditional probability distribution $q_{{\varvec{\phi }}} ({\varvec{z}} \mid {\varvec{x}}^{(i)})$ and taking the variational lower bound of the log-likelihood gives the following objective function:

11 $$\begin{aligned} \log p_{{\varvec{\theta }}} ( {\varvec{x}}^{(i)} )&\ge - D_{\text {KL}} {(} q_{{\varvec{\phi }}} ({\varvec{z}} \mid {\varvec{x}}^{(i)}) \Vert p({\varvec{z}}) ) + {\mathbb {E}}_{q_{{\varvec{\phi }}} ({\varvec{z}} \mid {\varvec{x}})} {[} \log p_{{\varvec{\theta }}}({\varvec{x}}^{(i)} \mid {\varvec{z}}) ] = {\mathcal {L}} ( {\varvec{\theta }}, {\varvec{\phi }}; {\varvec{x}}^{(i)} ).\end{aligned}$$

In the above equation, $p({\varvec{z}})$ is the prior distribution of latent variable ${\varvec{z}}$, and $D_{\text {KL}}(q\Vert p)$ is the Kullback–Leibler divergence^29^ of probability distributions *q* and *p*. The first term of the objective function corresponds to the regularization, and the second term corresponds to the reconstruction error. The VAE models conditional distributions $p_{{\varvec{\theta }}}({\varvec{x}}^{(i)} \mid {\varvec{z}})$ and $q_{{\varvec{\phi }}} ({\varvec{z}} \mid {\varvec{x}}^{(i)})$ using neural networks. To optimize parameters ${\varvec{\theta }}$ and ${\varvec{\phi }}$ by backpropagation, samples were generated using a method called *reparameterization trick* with encoder $q_{{\varvec{\phi }}} ({\varvec{z}} \mid {\varvec{x}}^{(i)})$. The latent variable is modeled as follows:

12 $$\begin{aligned} {\varvec{z}}&= g_{{\varvec{\phi }}} ({\varvec{\epsilon }}, {\varvec{x}}) = {\varvec{\mu }} + {\varvec{\sigma }} \odot {\varvec{\epsilon }}, \end{aligned}$$

to decompose ${\varvec{z}}$ into random variable ${\varvec{\epsilon }}$ and deterministic variables ${\varvec{\mu }}$ and ${\varvec{\sigma }}$, where $\odot$ indicates Hadamard–Schur product. Giving ${\varvec{\epsilon }}$ as a sample from the standard Gaussian distribution eliminates the need for a complicated integral during training. If the above conditions are assumed and the expected reconstruction error ${\mathbb {E}}_{q_{{\varvec{\phi }}} ({\varvec{z}} \mid {\varvec{x}})} {[} \log p_{{\varvec{\theta }}}({\varvec{x}}^{(i)} \mid {\varvec{z}}) ]$ is approximated by a sample average, Eq. (11) can be rewritten as follows:

13 $$\begin{aligned} {\mathcal {L}} ( {\varvec{\theta }}, {\varvec{\phi }}; {\varvec{x}}^{(i)} ) \simeq \frac{1}{2} \sum _{j=1}^{N_z} {(} 1 + \log {(} (\sigma _j^{(i)})^2 ) - (\mu _j^{(i)})^2 - (\sigma _j^{(i)})^2 ) + \frac{1}{L} \sum _{l=1}^L \log p_{{\varvec{\theta }}}({\varvec{x}}^{(i)} \mid {\varvec{z}}^{(i,l)}). \end{aligned}$$

${\varvec{\phi }}$ parameterizes the outputs of the encoder ${\varvec{\mu }}$ and ${\varvec{\sigma }}$. Both parameters ${\varvec{\theta }}$ and ${\varvec{\phi }}$ were trained by the gradient ascent method to maximize Eq. (13). The output of the decoder was set as the probability of the Bernoulli distribution, and the expectation of the conditional probability, namely, the second term of the objective function, was approximated by averaging *L* samples.

### Network architecture and optimization procedure

Since our research focuses on the behavior of VAE inference, we need to reduce dependence on network structure as much as possible. Based on this motivation, we used a separate three-layer, fully connected neural network for the encoder $q_{{\varvec{\phi }}} ({\varvec{z}} \mid {\varvec{x}}^{(i)})$ and decoder $p_{{\varvec{\theta }}}({\varvec{x}}^{(i)} \mid {\varvec{z}})$ mentioned above. The fully connected neural network is consists of a fully connected layer:

14 $$\begin{aligned} h^l_i = \text {activation} \left( \sum _{j=1}^{ {\# \text{units}}} W_{ij} h^{l-1}_j + b_j \right) . \end{aligned}$$

$h^l_i$ expresses the *i*-th unit (neuron) of the *l*-th layer, and ${W_{ij}}$ and ${b_{j}}$ are the parameters of each layer. The input to the first layer of the encoder ${\varvec{h}}^0 = {[}\cdots , h^0_j, \cdots ]^\top$ corresponds to a data point ${\varvec{x}}$, and the output of the encoder ${\varvec{h}}^2$ corresponds to ${\varvec{\mu }}$ and $\log {\varvec{\sigma }}^2$ in Eqs. (11)and (13). Likewise, the input and the output of the decoder correspond to ${\varvec{z}}$ and ${\varvec{x}}$, respectively. The number of units in the middle layer was set to 1,024, and the activation function was set as tanh:

15 $$\begin{aligned} \tanh (x) = \frac{e^{2x} - 1}{e^{2x} + 1}. \end{aligned}$$

**Figure 10:**
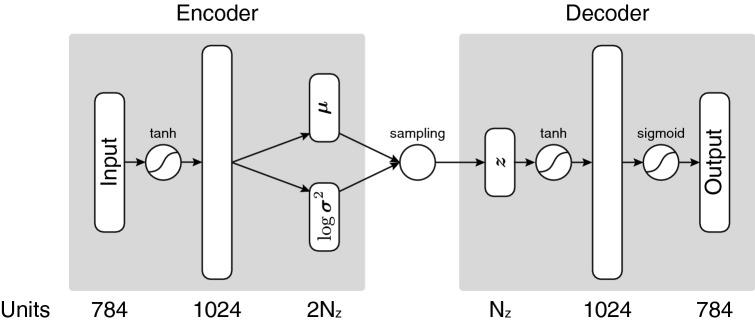
Network architecture.

We used the sigmoid function

16 $$\begin{aligned} \text {sigmoid}(x) = \frac{1}{1 + e^{-x}} \end{aligned}$$

for the activation function of the decoder’s last layer to normalize the output of the model to [0, 1]. The number of units of latent variable $N_z$ was set to 100 unless otherwise noted. We show the schematic diagram of the network architecture in Fig. 10.

We used Adam^30^ as the parameter optimization algorithm. Adam updates the model parameter $\theta$ according to the objective function *f* with the following equations:

17 $$\begin{aligned} g_t \leftarrow&{\varvec{\nabla }}_\theta f_t(\theta _{t - 1}), \end{aligned}$$

18 $$\begin{aligned} m_t \leftarrow&\beta _1 \cdot m_{t - 1} + (1 - \beta _1) \cdot g_t, \end{aligned}$$

19 $$\begin{aligned} v_t \leftarrow&\beta _2 \cdot v_{t - 1} + (1 - \beta _2) \cdot g_t^2, \end{aligned}$$

20 $$\begin{aligned} {\hat{m}}_t \leftarrow&m_t / (1 - \beta _1^t), \end{aligned}$$

21 $$\begin{aligned} {\hat{v}}_t \leftarrow&v_t / (1 - \beta _2^t), \end{aligned}$$

22 $$\begin{aligned} \theta _t \leftarrow&\theta _{t - 1} - \alpha \cdot {\hat{m}}_t / \left( \sqrt{{\hat{v}}_t} + \epsilon \right) . \end{aligned}$$

The lower script *t* in the above equations expresses the time step of the optimization. We note that this time step is for the training phase and is not for the inference phase. $$g, m, v, {\hat{m}}$$, and $${\hat{v}}$$ are the intermediate variables to compute the parameter $$\theta$$ of the next time step. $$\alpha$$, $$\beta _1$$, $$\beta _2$$, and $$\epsilon$$ in the above equations are the hyperparameters for the optimization. $$\alpha$$ is the learning rate or step size of the optimization. $$\beta _1$$ and $$\beta _2$$$$\in [0, 1)$$ control the exponential decay rates of moving averages. We set $$\beta _1$$, $$\beta _2$$, and $$\epsilon$$ to the default values 0.9, 0.999, and $$10^{-8}$$. The learning rate $$\alpha$$ was reduced in descending order as follows: 0.0005, 0.0001, and 0.00005. In our setup, we used the variational lower bound of the log-likelihood (Eq. (13)) as the objective function, and the parameters of the encoder $${\varvec{\phi }}$$ and the decoder $${\varvec{\theta }}$$ are the target to optimize. We set the number of samples for calculating the reconstruction error to $$L=2$$. We trained the VAE against the MNIST dataset for 1,500 epochs. The MNIST dataset consists of $$28 \times 28$$-pixels ‘0’–‘9’ handwritten images with 60,000 training data and 10,000 test data. These data are considered to have cluster structures consisting of 10 types of labels, namely, ‘0’–‘9’.

### Inference procedure and noise injection

Here, we describe how to perform repeated inference. In this study, noisy MNIST data were inferred using the trained network according to the following procedure, and the time evolution of latent variable ${\varvec{z}}(t)$ was obtained. First, noise was added to an image of the training dataset. Pixels with probability *p* were selected from 784 pixels, the image intensities of the selected pixels were swapped, and the image was set as ${\varvec{x}}_0$. Next, the data variable in step $t=0$ was taken as ${\varvec{x}}(0)={\varvec{x}}_0$. Finally, generation and recognition were repeated *T* times according to the following two equations:

23 $$\begin{aligned} {\varvec{x}}(t+1)&= {\mathbb {E}}_{ p_{{\varvec{\theta }}}({\varvec{x}} \mid {\varvec{z}}(t)) } {[} {\varvec{x}} ], \end{aligned}$$

24 $$\begin{aligned} {\varvec{z}}(t)&= {\mathbb {E}}_{ q_{{\varvec{\phi }}}({\varvec{z}} \mid {\varvec{x}}(t)) } {[} {\varvec{z}} ], \end{aligned}$$

to obtain the time evolutions of data variable ${\varvec{x}}(t)$ and latent variable ${\varvec{z}}(t)$. We modeled the observation process by an independent Bernoulli distribution, so the output of the decoder corresponds to the expected value of ${\varvec{x}}$:

25 $$\begin{aligned} {\varvec{x}}(t+1) = {\mathbb {E}}_{ p_{{\varvec{\theta }}}({\varvec{x}} \mid {\varvec{z}}(t)) } {[} {\varvec{x}} ] = \text {decoder}_{{\varvec{\theta }}}({\varvec{z}}(t)). \end{aligned}$$

Also, since we used the Gaussian encoder as we mentioned above, the first half of the output of the encoder (${\varvec{\mu }}$) is the expected value of ${\varvec{z}}$. Therefore,

26 $$\begin{aligned} {\varvec{z}}(t) = {\mathbb {E}}_{ q_{{\varvec{\phi }}}({\varvec{z}} \mid {\varvec{x}}(t)) } {[} {\varvec{z}} ] = {\varvec{\mu }}_{{\varvec{\phi }}}({\varvec{x}}(t)). \end{aligned}$$

We expressed the first half of the encoder’s output as ${\varvec{\mu }}_{{\varvec{\phi }}}(\cdot )$. We note that we do not need any approximation to compute the expected values thanks to the model definition. The dynamics of ${\varvec{x}}(t)$ and ${\varvec{z}}(t)$ were numerically analyzed. The randomness of these dynamics ${\varvec{x}}(t)$ and ${\varvec{z}}(t)$ only comes from the randomness of the input ${\varvec{x}}(0)$.

The deep learning framework Keras^31^ version 2.0.2 on Theano^32^ backend version 0.9.0, running on CUDA 8.0 with CuDNN v5.1 on NVIDIA Tesla K80, was used for all numerical simulations.

## Supplementary information

Supplementary Information 1.

Supplementary Video S1.

Supplementary Video S2.

Supplementary Video S3.

Supplementary Video S4.

Supplementary Video S5.

Supplementary Video S6.

Supplementary Video S7.

Supplementary Video S8.

Supplementary Video S9.

Supplementary Video S10.
