# Supplementary material for: Collective dynamics of repeated inference in variational autoencoder rapidly find cluster structure
Source: Sci Rep. 2020 Sep 29;10:16001. doi: 10.1038/s41598-020-72593-4 (PMC7524732; doi:10.1038/s41598-020-72593-4)
Supplement: Supplementary file 1 — Supplementary Information 1. [file 41598_2020_72593_MOESM1_ESM.pdf]

Supplementary Information:

Collective Dynamics of Repeated Inference in Variational Autoencoder Rapidly Find Cluster Structure

Yoshihiro Nagano[1,2], Ryo Karakida[3], and Masato Okada[1,3,*]

[1]The University of Tokyo, Department of Complexity Science and Engineering, Chiba, 277-8561, Japan
[2]Research Fellow of the Japan Society for the Promotion of Science, Tokyo, 102-0083, Japan
[3]National Institute of Advanced Industrial Science and Technology, Artificial Intelligence Research Center, Tokyo, 135-0064, Japan
[*]okada@edu.k.u-tokyo.ac.jp

A: Consecutive Samples for MNIST Dataset

We showed the consecutive samples for the initial image of '6' in the main text. In this section, we additionally show the consecutive samples for other initial images in fig. S1. The visualizations and the experimental conditions all follow the one of the main text. From the figures, the noise of the generated images gradually decreased with the inference step. In fig. S1, we note that we visualized the trials where the generated image at the last inference step clearly remained at the same label. The result of repeated inferences at the final step varies stochastically due to the effect of noise applied to the initial value. To clarify this effect, we also show the trials where the trained model failed to infer the appropriate images in fig. S2. The generated images tended to transition to perceptually similar images to the initial images when the inference fails: '2' to '8' or '5' to '3'. We also verified the effect of such a type of failure on the distance from the concept vector in a later section. Please see these sections for more detail

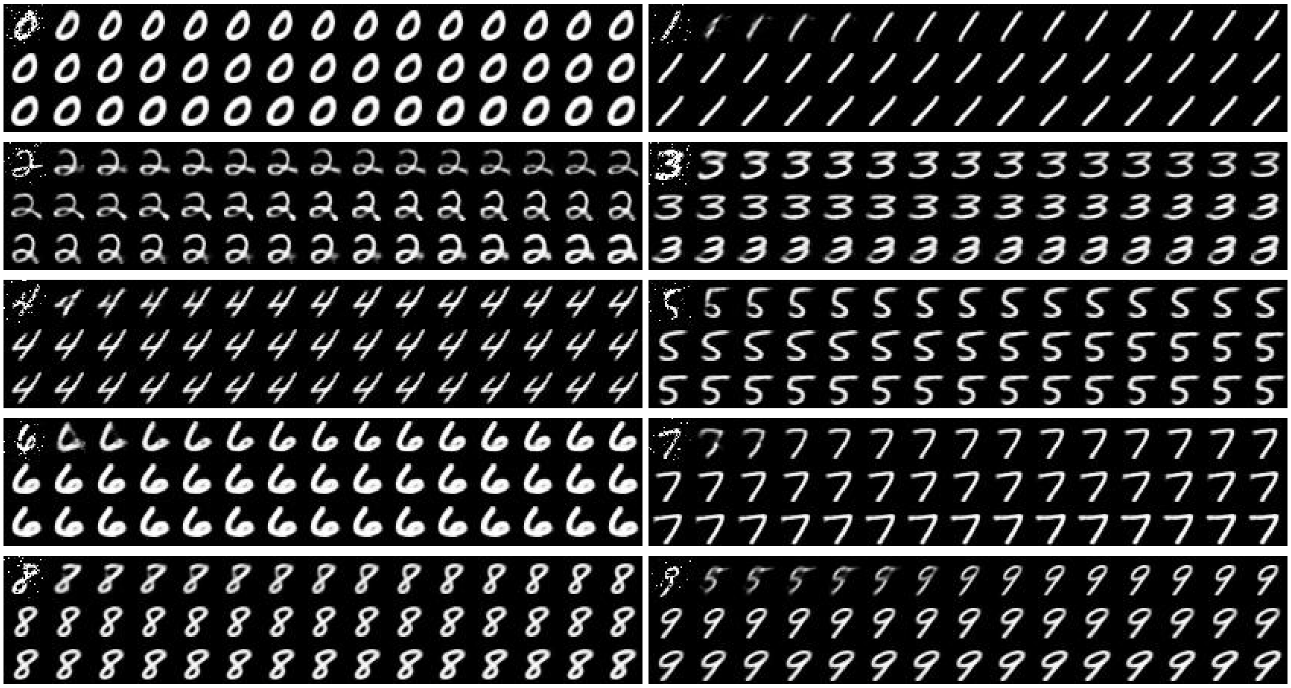

Figure S1. Consecutive samples for the MNIST dataset. All figures only show the trials where the generated image at the last inference step clearly remained at the same label.

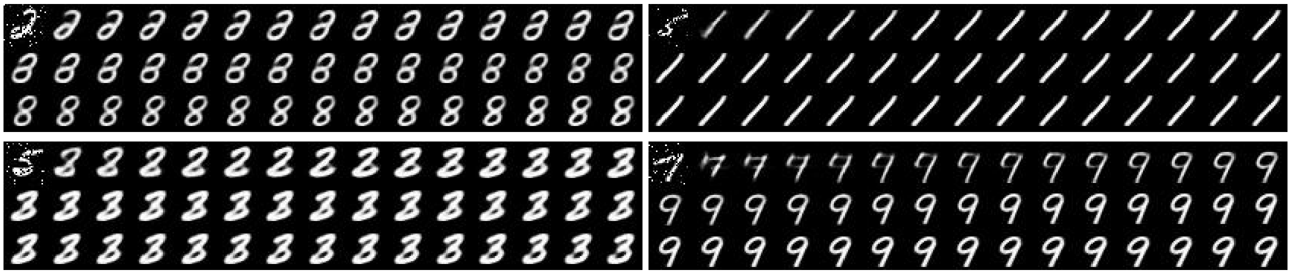

Figure S2. Consecutive samples for the MNIST dataset. All figures show the trials where the trained model failed to infer the appropriate images.

B: Visualization of Inferred Images in the Latent Space

In the main text, we visualized the activity patterns for the initial images of '1' during repeated inferences by using PCAs. These visualizations numerically clarified that one large crowd of these activity patterns quickly branched into two clusters during inferences. We also showed the perceptually generated images during these processes in this section.

Figure S3 is the visualization of the perceptually generated images during the repeated inferences mentioned in the main text. Each figure (from fig. S3a to fig. S3e) corresponds to the two-dimensional PCA embeddings in the main text. The x- and the y-axes represent the first and the second principal components. At every time step t, the activity pattern $\boldsymbol{z}^{(i)}(t)$ for i-th initial image specifies the coordinate in the principal component space. We plotted the inferred images $\mathbb{E}_{p_{\boldsymbol{\theta}}(\boldsymbol{x}|\boldsymbol{z}^{(i)}(t))}[\boldsymbol{x}]$ for the latent activity patterns in these coordinates. We cropped the x- and the y-axes in the range of $[-2,2]$ for clarity.

At the initial phase (fig. S3a and fig. S3b) of repeated inferences, the inferred images moderately included some noise. At the time step $t = 10$ (fig. S3c), the crowd of the activity patterns branched into two clusters. We found that the upper half of these two clusters corresponded to the trials where the inferred images deviated from '1'. The lower half of the cluster shaped like a string expressed various angles of '1' smoothly. The angle of the inferred image of '1' gradually shifted from left to right. These continuously distributed angles converged to several specific values at the end of the inference (fig. S3e). We also showed the latent activity patterns for the initial images of all labels in the movie format. The crowd of these activity patterns branched to multiple string-shaped clusters and slowly converged to points. These results suggest that there are multiple saddle points, which have stable and unstable directions, in the latent space of trained VAEs. It is indicated that the data points which typically appear in the training dataset became stable fixed points and the lines between these points appeared as string-shaped subspaces.

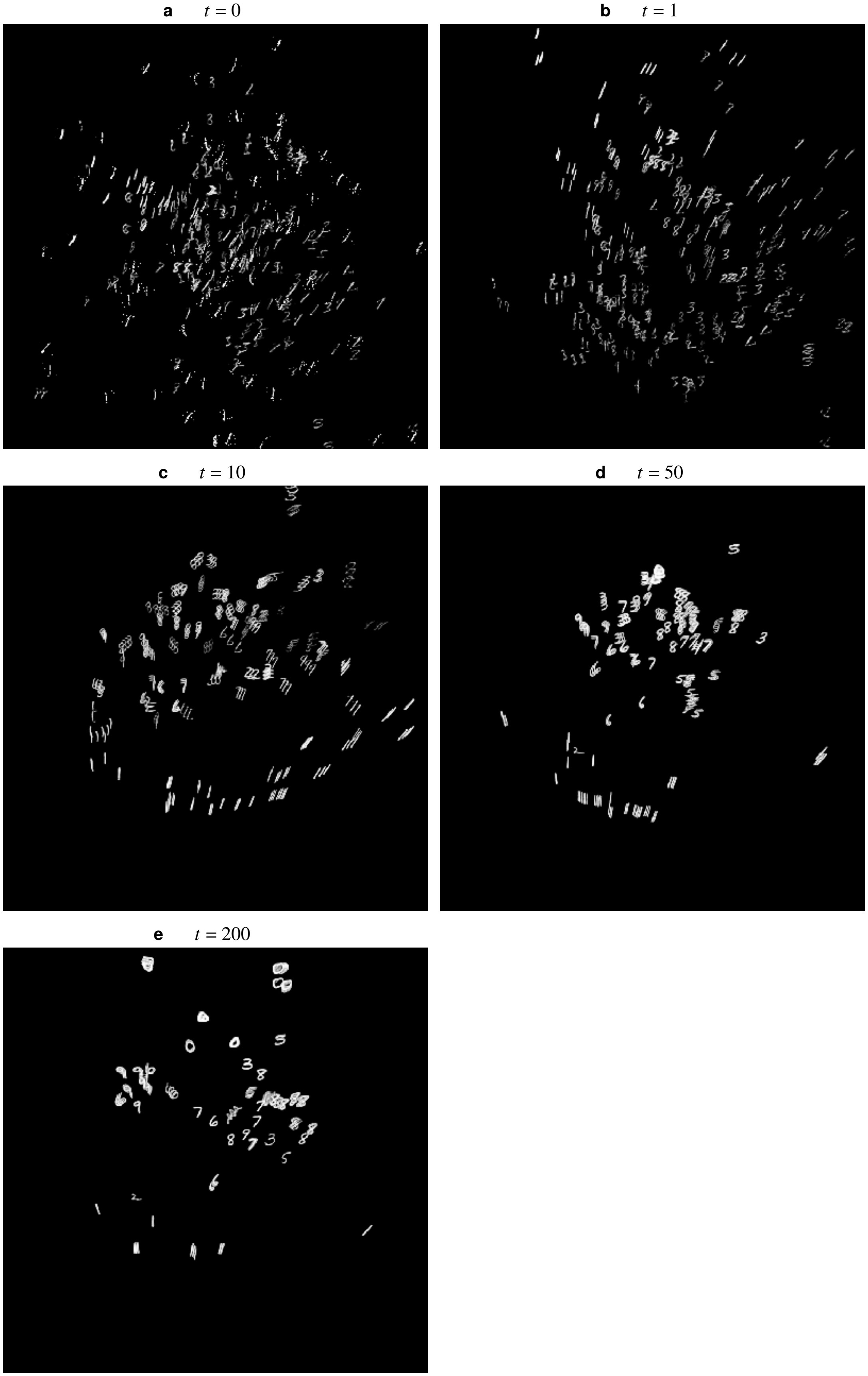

Figure S3. (a–e): The inferred images that correspond to the dynamics of activity patterns in latent space. The x- and y-axes indicate the first and the second principal components. Each figure shows the snapshot of the activity patterns at the specific time step from $t = 0$ to $t = 200$. The x- and the y-axes are cropped in the range of $[-2, 2]$ for clarity.

C: Additional Analyses of the Trajectory of Latent Activity Patterns

In the main text, we examined the time development of distances between the activity patterns and the concept/memory vectors. These results revealed that the distance to the memory vector behaves qualitatively similar to the distance to the concept vector. Here, we show another metric to clarify where the activity patterns was going.

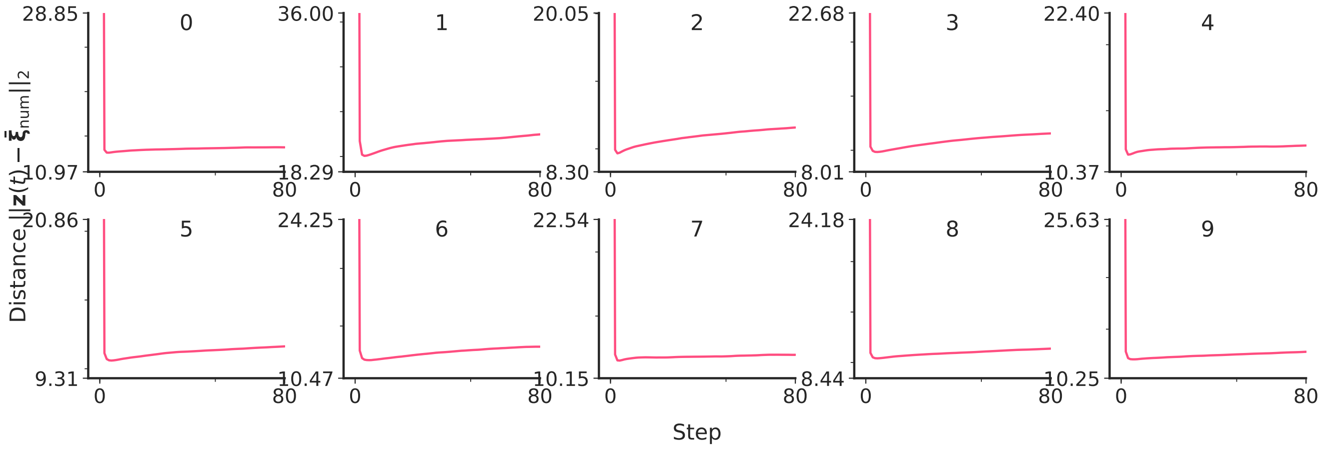

Figure S4. Time development of the Mahalanobis distance between the latent activity patterns and the concept vector. The experimental setting is as same as for the Euclid distance.

We also verified the time development of Mahalanobis distance from the concept vector to clarify that approach to the concept vector is not due to non-spherical clusters. Figure S4 shows the time development of Mahalanobis distance $\sqrt{(z(t)-\bar{\xi}_{\text{num}})^\top \Sigma_{\text{num}}^{-1}(z(t)-\bar{\xi}_{\text{num}})}$. The result was qualitatively similar to the Euclidean distance. The increase in the distance at the end of the inference was less significant than in Euclid distance. This result indirectly suggests that the activity patterns wander around the cluster of each cluster.

D: Numerical Evaluations on Fashion-MNIST dataset

We numerically analyzed the collective behavior of latent activity patterns using the MNIST dataset in the main text. This section shows the same numerical experiment on the Fashion-MNIST dataset, which is a dataset of Zalando's article images consisting of various fashion images. The Fashion-MNIST dataset was created to replace the original MNIST dataset for benchmarking machine learning algorithms. The size of images and the number of labels are exactly the same as the MNIST dataset. Based on this construction, the Fashion-MNIST dataset is also considered to have a cluster structure. We used the same network architecture and the hyperparameters as the experiments on the MNIST in the following.

Both fig. S5 and fig. S6 are the consecutive samples for the Fashion-MNIST dataset. Figure S5 shows the trials where the trained VAEs succeeded to infer the original label at the final step of repeated inferences, and fig. S6 shows the trials where the inference failed. The VAEs succeeded in removing the noise in the initial images as well as the case of the MNIST dataset. Notably, they reduced the noise drastically during the first several steps of repeated inferences. Fine structures such as the design of T-shirts were lost. Such behavior is considered to occur due to the limitation of multilayer perceptrons' ability to express and the noise applied to the initial image. In trials where inference has failed, the trained models inferred the images that only preserve the rough structure in the initial images. For example, the detailed structure of the handle of the bag was lost and became a jacket-like image. The lower half of the pants was integrated and changed to a dress-like image.

Then, we analyzed the distance between the latent activity patterns and the cluster centers. Figure S7 shows the time development for all labels. The meaning of the figure is as same as in the main text. The distance between the cluster center and 300 different initial images was calculated. Each figure corresponds to each label that was used as initial input for the VAE. The x-axis expresses the time step t of repeated inference, and the y-axis expresses the Euclidean distance. All results were qualitatively consistent with the results of the MNIST dataset. The activity patterns in the latent space quickly approached the cluster centers and slowly left.

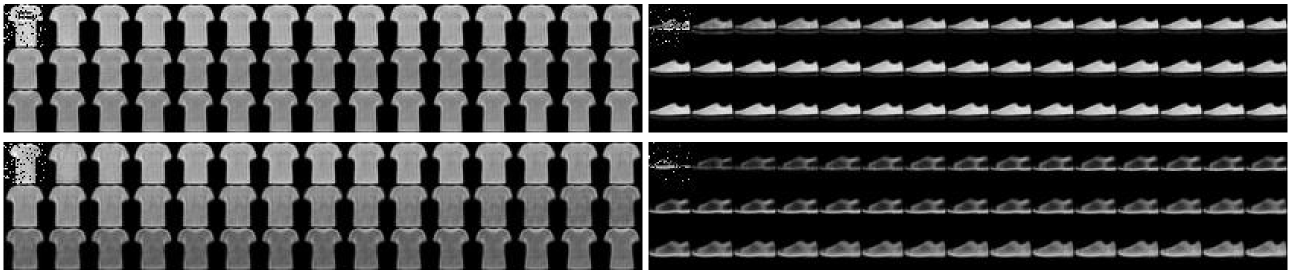

Figure S5. Consecutive samples for the Fashion-MNIST dataset. All figures only show the trials where the generated image at the last inference step clearly remained at the same label.

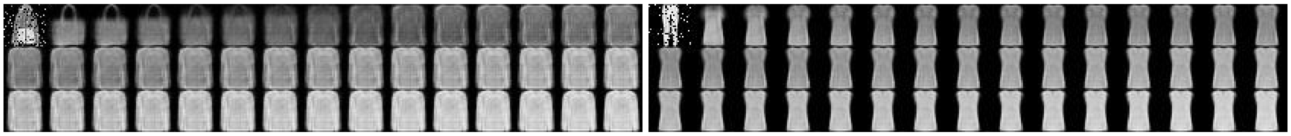

Figure S6. Consecutive samples for the Fashion-MNIST dataset. All figures show the trials where the trained model failed to infer the appropriate images.

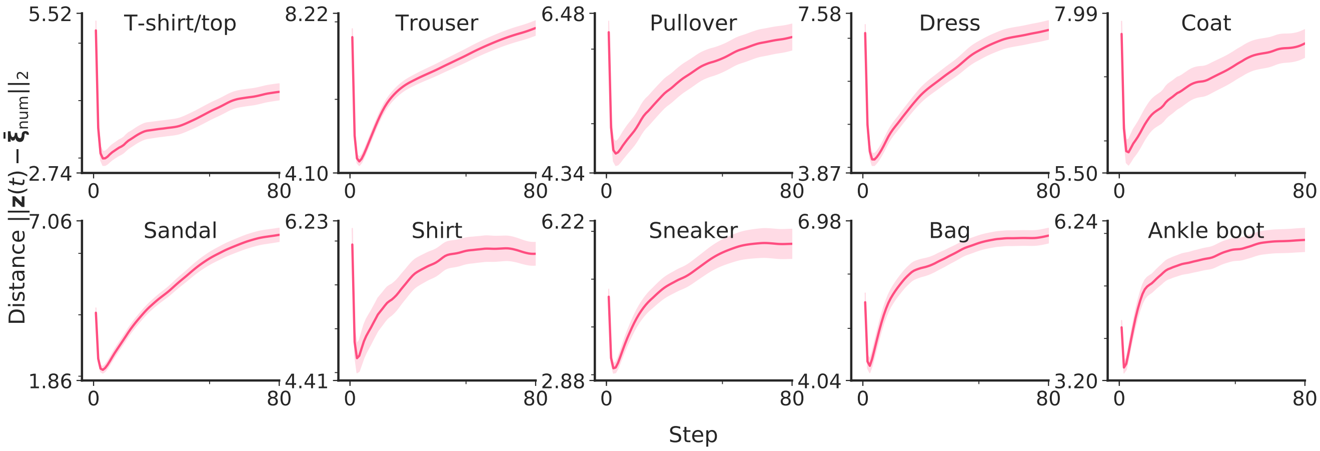

Figure S7. Time development of the distance from $\bar{\boldsymbol{\xi}}_{\mathrm{num}}$ for all labels of the Fashion-MNIST data. The shades represent the ±1 standard error of the mean (300 trials). All figures were generated with the noise fraction $p = 0.2$.

E: Detailed Analysis of the Orthogonality of the Cluster Centers

In this section, we show the detailed change in the cosine similarity matrix C and the dynamics of the repeated inferences according to the latent space dimensionality N_z.

Figures S8a to S8e visualize the cosine similarity matrices for corresponding latent space dimensionality N_z. The x- and the y-axes indicate the row and column of the matrix, and the figures visualize the value of each element as a heatmap. Because the cosine similarity matrix is symmetric, we omitted the upper half of the figures. The number of neurons in the latent variable N_z was controlled in the following order: 2, 5, 10, 20, and 100, and we trained the VAEs from scratch for each hyperparameter. By definition, the value of the diagonal elements is one. In the main text, we showed that the off-diagonal terms of these matrices are globally large when the latent dimension is low, and are roughly the lowest at approximately $N_z = 10$ to 20 and remain the lowest value after that. According to figs. S8a to S8e, the values between structurally similar labels such as '0' and '6' or '7' and '9' were large. The cosine similarity between these values remained high even for the large N_z. We also compared the time evolution of the distance from a cluster center of '6' with different model hyperparameters in figs. S8f to S8j. Under condition $N_z = 5$, the trajectory escaped the cluster center, and the trajectory also did not approach the cluster center.

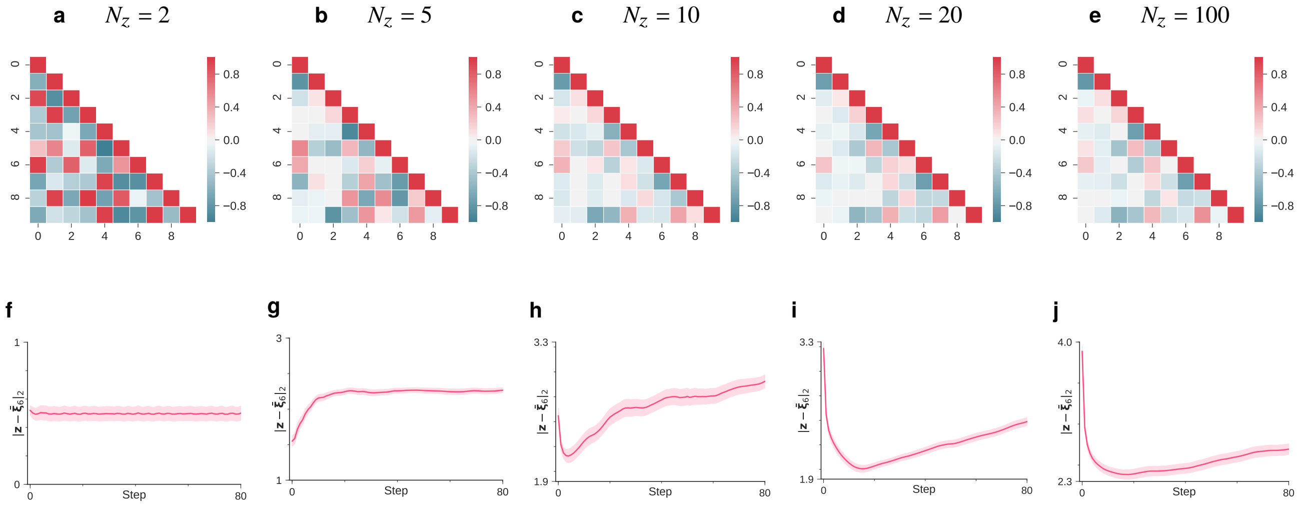

Figure S8. (a–e): Cosine similarity between the memory patterns of each concept. The number of elements in the latent variable is written as N_z. (f–j): Time development of the distance with a concept of '6' in (a–e).

F: Verifying the Effect of Moving Other Numbers

The possibility that other labels confused the repeated inferences in the VAE was numerically tested. It is conceivable that the escape from the cluster center is caused by attraction to another cluster. To eliminate this possibility, a discriminative neural network was constructed separately from the VAE, and the final state of inference of the VAE was classified.

In the following analysis, a model with an Input-Convolution-Convolution-Pooling-Dropout1-FullyConnected-Dropout2-SoftMax structure was constructed as the discriminative neural network. The kernel size of the convolution was set to three, the pooling size was set to two, and the dropout probability was set to 0.25, 0.5 in order from the input side. A rectified linear unit (ReLU) was used as the activation function. This model recorded a discrimination ability of 99.25% against the test data included in the MNIST dataset.

The result of classifying the final state of inference using the aforementioned discriminative neural network is shown in fig. S9a. The x-axis represents a trial of each inference with various initial images, and the y-axis represents the number label. The heat map indicates the classification probability for each number label. An image of '6' was used as the initial value of the inference. The discriminator classified the final state of 193 of 300 trials as '6'.

We considered the effect of other labels as the cause of the neural activity patterns approaching mismatched clusters. Using the label '6', we first measured the distances between each neural activity pattern and the concept of '6'. We divided all the neural activity patterns into two conditions. We classified the trial in which the final state of the trajectory was inside the cluster of '6', as a condition "only 6", and all trials as a condition "all". Then, we averaged the distances in each condition and

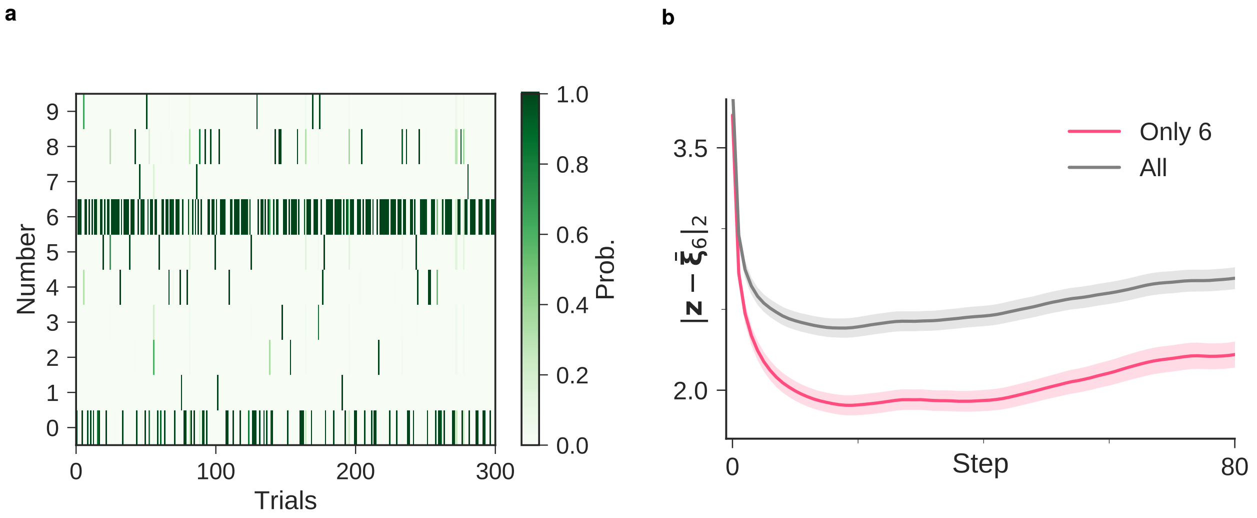

Figure S9. (a) The result of classifying the final state $T = 80$ of the inference for image '6'. (b) The time evolution of the distance from a concept of '6'. The condition excluding trials in which the activity pattern switched to different numbers is expressed in red, and the condition containing all the trials is expressed in gray.

compared their means. The average trajectories are compared in fig. S9b. The red shows the average of the trial with the final state identified as '6', and gray shows the average of all trials.

As shown in fig. S9b, the neural activity patterns in both conditions approached the cluster center before moving to the corresponding patterns. This result suggests that the presence of other labels does not cause the neural activity patterns to move away from the cluster center.
